# PAT-H-MS coupled with laser microdissection to study histone post-translational modifications in selected cell populations from pathology samples

**DOI:** 10.1186/s13148-017-0369-8

**Published:** 2017-07-11

**Authors:** Roberta Noberini, Rémi Longuespée, Cristina Richichi, Giancarlo Pruneri, Mark Kriegsmann, Giuliana Pelicci, Tiziana Bonaldi

**Affiliations:** 1 Center for Genomic Science of IIT@ SEMM, Istituto Italiano di Tecnologia, Via Adamello 16, 20139 Milan, Italy; 2 Institute of Pathology, University of Heidelberg, Im Neuenheimer Feld 224, 69620 Heidelberg, Germany; 3 Department of Experimental Oncology, European Institute of Oncology, Via Adamello 16, 20139 Milan, Italy; 4 Biobank for Translational Medicine Unit, Department of Pathology, European Institute of Oncology, Via Ripamonti 435, 20141 Milan, Italy; 5 School of Medicine, University of Milan, 20122 Milan, Italy; 6 Department of Translational Medicine, Piemonte Orientale University “Amedeo Avogadro”, 28100 Novara, Italy

**Keywords:** Histone post-translational modifications, PAT-H-MS, Laser microdissection, Proteomics, Epigenetic marker, Formalin-fixed paraffin embedded, Mass spectrometry

## Abstract

**Background:**

Aberrations in histone post-translational modifications (hPTMs) have been linked with various pathologies, including cancer, and could not only represent useful biomarkers but also suggest possible targetable epigenetic mechanisms. We have recently developed an approach, termed pathology tissue analysis of histones by mass spectrometry (PAT-H-MS), that allows performing a comprehensive and quantitative analysis of histone PTMs from formalin-fixed paraffin-embedded pathology samples. Despite its great potential, the application of this technique is limited by tissue heterogeneity.

**Methods:**

In this study, we further implemented the PAT-H-MS approach by coupling it with techniques aimed at reducing sample heterogeneity and selecting specific portions or cell populations within the samples, such as manual macrodissection and laser microdissection (LMD).

**Results:**

When applied to the analysis of a small set of breast cancer samples, LMD-PAT-H-MS allowed detecting more marked changes between luminal A-like and triple negative patients as compared with the classical approach. These changes included not only the already known H3 K27me3 and K9me3 marks, but also H3 K36me1, which was found increased in triple negative samples and validated on a larger cohort of patients, and could represent a potential novel marker distinguishing breast cancer subtypes.

**Conclusions:**

These results show the feasibility of applying techniques to reduce sample heterogeneity, including laser microdissection, to the PAT-H-MS protocol, providing new tools in clinical epigenetics and opening new avenues for the comprehensive analysis of histone post-translational modifications in selected cell populations.

**Electronic supplementary material:**

The online version of this article (doi:10.1186/s13148-017-0369-8) contains supplementary material, which is available to authorized users.

## Background

Histone post-translational modifications (hPTMs) generate a complex combinatorial code that is crucial to regulate gene expression and determine cell fate [1]. Increasing evidence has linked aberrations in hPTMs with various pathologies, including cancer, suggesting that they could represent useful biomarkers for patient stratification. Indeed, after the landmark discoveries of the loss of H4-lysine 16 acetylation (H4K16ac) and H4-lysine 20 trimethylation (H4K20me3) in cancer [2], and of the prognostic value of hPTMs in various types of cancers [3, 4], many more histone marks have been recognized as possible biomarkers in different diseases, and particularly in cancer [5]. In addition, because changes in hPTM levels are usually the result of the aberrant expression or mislocalization of histone modifying enzymes [6], profiling histone modification in disease could not only help uncover possible epigenetic mechanisms underlying different pathologies but also provide novel epigenetic pathways targetable for therapy. Indeed, since epigenetic changes, unlike genetic ones, are intrinsically reversible and can be overturned, epigenetic therapies are a promising avenue in translational research.

In recent years mass spectrometry (MS) has emerged as a powerful method to analyze hPTMs, thanks to its accuracy, its unbiased nature, and its ability to accurately quantify modifications and their combinations [7], which represent important advantages over traditional antibody based-methods. However, the potential offered by the MS-based analysis of hPTMs in clinical cancer samples has been left largely unexploited. Indeed, most of the studies employing MS-based techniques, which can provide a comprehensive and quantitative view on hPTM patters, have focused on cell lines and animal tissue, while clinical samples are usually tested through antibody-based techniques.

Recently, we have reported for the first time a method that allows the MS-based analysis of hPTMs from human pathology tissues, termed pathology tissue analysis of histones by mass spectrometry (PAT-H-MS) [8], which combines protocols used for global proteomic studies of formalin-fixed paraffin-embedded (FFPE) tissues [9] with a proteomic workflow optimized for hPTM analysis [10]. By using this method, we revealed significant changes in histone H3 methylation patterns between luminal A-like and triple negative breast cancer subtypes. By combining the power of the MS-based analysis of hPTMs with the enormous amount of clinical information contained in FFPE archives, which represent the storage method of choice for clinical specimens, PAT-H-MS is a significant technological advancement in clinical epigenetics.

However, a limitation of this approach, which is shared by any other application in which FFPE sections are used as starting material, is tissue heterogeneity. This is a particular issue for tumor specimens, which often contain nontumoral cells that can mask or reduce the contribution of the tumor cells. In this study, we implemented the PAT-H-MS approach by coupling it with techniques aimed at reducing sample heterogeneity and selecting specific portions or cell populations within the samples, such as manual macrodissection and laser microdissection (LMD), and show the potential of LMD-PAT-H-MS by applying it to a small panel of breast cancer luminal A-like and triple negative samples.

## Methods

### Collection of specimens and preparation of FFPE tissues

Spleen tissue was collected from a leukemic mouse with splenomegaly, washed in PBS and incubated for 16 h at room temperature in a 4% paraformaldehyde solution. The fixed spleen was then routinely dehydrated with increasing concentrations of ethanol (70, 80, 90, and 100%) and subsequently included in paraffin using a tissue processor (Leica ASP300).

Glioblastoma (GBM) specimens were collected from patients at the Department of Neurosurgery at Istituto Neurologico Carlo Besta. Human GBM-derived neurospheres were obtained and grown as previously described [11]. Prior to injection in mice, the neurospheres were mechanically dissociated, and the cells were resuspended in 2 μL of phosphate-buffered saline and stereotaxically injected into the nucleus caudatus (1 mm posterior, 3 mm left lateral, 3.5 mm in depth from bregma) of 5-week-old female nu/nu CD1 mice (Charles River, Wilmington, MA). Whole mouse brains were collected and processed as described above.

Breast cancer frozen and FFPE specimens were obtained from patients with duct invasive carcinoma at the European Institute of Oncology, who were subjected to mastectomy or breast conserving surgery. FFPE resection specimens of duct invasive breast carcinoma for LMD were selected from the archive of the Institute of Pathology Heidelberg with the support of the National Center for Tumor Diseases (NCT, Heidelberg, Germany). Tumor cellularity was evaluated histologically, and the assessment of hormone and Her-2 receptors and the Ki-67 labeling index was performed as previously described [8]. Luminal A-like and triple negative subtypes were defined as follows: luminal A-like: ER and/or PgR(+), HER2(−), Ki67 < 20%; triple negative: ER, PgR, and HER2(−), irrespective of Ki67 score.

### PAT-H-MS

Histones were isolated from FFPE tissues as recently described [8]. Briefly, four 10-μm tissue sections were deparaffinized with four washes in hystolemon (Dasit Group Carlo Erba) and rehydrated from 100% ethanol to water (through intermediate incubations in ethanol 95, 70, 50, and 20%). Tissue samples were resuspended in 200 μL of 20 mM Tris pH 7.4 containing 2% SDS and were homogenized by sonication in a Branson Digital Sonifier 250 with a 3-mm microtip. Proteins were then extracted and de-crosslinked at 95 °C for 45 min and 65 °C for 4 h. The amount of histones was estimated by sodium dodecyl sulphate-polyacrylamide gel electrophoresis (SDS-PAGE) gel following protein detection with colloidal Comassie staining (Expedeon) by comparison with known amounts of recombinant histone H3.1 (New England Biolabs). For PAT-H-MS coupled with manual macrodissection or laser microdissction, 10-μm sections were placed on glass slides (Leica Microsystems) and deparaffinized by incubation in xylene (Carlo Erba, Milan, Italy) for 1 min. Tissue sections were subsequently rehydrated in decreasing concentrations of ethanol (100, 95, and 75%) and rinsed in deionized water for 30 s. The slides were stained with hematoxylin for 2 min, washed in deionized water, and dehydrated by incubation in 75% ethanol for 30 s. Each step was performed at room temperature. When using manual macrodissection, the tissue areas corresponding to the tumor xenografts and the normal mouse brain were morphologically evaluated by visualization under a microscope. The two areas, which were clearly identifiable macroscopically, were scraped off the slide with a scalpel into an eppendorf tube, washed once with 1 ml of histolemon to remove any remaining paraffin and rehydrated by a 3 min incubation at room temperature in decreasing concentrations of ethanol (100, 70, 50, 20, and water) followed by a 3 min centrifugation. The tissue was then processed as in the original PAT-H-MS protocol.

### PAT-H-MS coupled with LMD

Cancer areas were collected in Eppendorf tubes from tissue sections by laser microdissection, using a Leica LMD 7000 instrument (Leica Microsystems, Wetzlar, Germany) in the “draw and cut” mode with the following laser settings: wavelength 349 nm, pulse energy 2 μJ, numerical aperture 55, speed 15, specimen balance 35, head current 100%, pulse frequency 5000 Hz, and focus offset 65. Tissue pieces were transferred at the bottom of the tubes through a 3-min centrifugation at maximum speed. Tubes were opened carefully to avoid losing tissue pieces and were processed as described above for macrodissected tissue.

### Super-SILAC

A histone-focused super-stable isotope labeling by amino acid in cell culture (SILAC) approach was used as we have recently described [12]. MDA-MB-231, MDA-MB-468, MDA-MB-453, and MDA-MB-361 breast cancer cells lines were grown in SILAC-DMEM (Euroclone) supplemented with 2 mM l-glutamine, 146 mg/l of lysine (Sigma-Aldrich), 84 mg/l l-^13^C_6_^15^N_4_-arginine (Arg-10, Sigma-Aldrich), 10% dialyzed serum (Life Technologies), and penicillin/streptomycin for at least eight doublings to obtain complete labeling with heavy-labeled aminoacids. Histones were isolated from the different cell lines through nuclei isolation on a sucrose cushion followed by acidic extraction, as described [13], mixed in equal amounts, lyophilized, and stored at −80 °C until use.

### Histone digestion

About 2–5 μg of histones per run per sample were separated on a 17% SDS-PAGE gel and bands corresponding to histone H3 were excised and in-gel digested as previously described [13]. Briefly, gel bands were cut in pieces and de-stained with repeated washes in 50% acetonitrile (ACN) in H_2_O, alternated with dehydration steps in 100% ACN. Gel pieces were then in-gel chemically alkylated with D_6_-acetic anhydride (Sigma-Aldrich) 1:9 in 1 M NH_4_HCO_3_, using CH_3_COONa as catalyzer. After shaking for 3 h at 37 °C, chemically modified gel slices were washed with NH_4_HCO_3_, alternated with ACN at increasing percentages (from 50 to 100). The in-gel digestion was performed overnight with 100 ng/μL trypsin (Promega) in 50 mM NH_4_HCO_3_ at 37 °C, in order to obtain an “Arg-C like” in-gel digestion that originates histone peptides of optimal length for MS analysis by cleaving at the C-terminal of arginine residues. Finally, digested peptides were extracted using 5% formic acid alternated with ACN 100%. In SILAC experimental set-ups, unlabeled and heavy-labeled histones were mixed in equal amounts prior to gel separation, and then processed as described above. Digested peptides were desalted and concentrated using a combination of reversed-phase C_18_/C and strong cation exchange (SCX) chromatography on handmade nanocolumns (StageTips). Digested peptides were then eluted with 80% ACN/0.5% acetic acid and 5% NH_4_OH/30% methanol from C_18_/C and SCX StageTips, respectively. Eluted peptides were lyophilized, resuspended in 1% TFA, pooled, and subjected to LC-MS/MS analysis.

### LC-MS/MS

Peptide mixtures were separated by reversed-phase chromatography on an in-house-made 25-cm column (inner diameter 75 μm, outer diameter 350 μm, outer diameter 1.9 μm ReproSil, Pur C18AQ medium), using an ultra-nanoflow high-performance liquid chromatography (HPLC) system (EASY-nLC™ 1000, Thermo Fisher Scientic) or an EASY-Spray column (Thermo Fisher Scientic), 50 cm (inner diameter 75 μm, PepMap C18, 2 μm particles), which were connected online to a Q Exactive HF instrument (Thermo Fisher Scientific) through a Nanospray Flex™ or an EASY-Spray™ Ion Sources (Thermo Fisher Scientific), respectively. Solvent A was 0.1% formic acid (FA) in ddH_2_O and solvent B was 80% ACN plus 0.1% FA. Peptides were injected in an aqueous 1% TFA solution at a flow rate of 500 nl/min and were separated with a 100-min linear gradient of 0–40% solvent B, followed by a 5-min gradient of 40–60% and a 5-min gradient of 60–95% at a flow rate of 250 nl/min. The Q Exactive HF instrument was operated in the data-dependent acquisition (DDA) mode to automatically switch between full-scan MS and tandem mass spectrometry (MS/MS) acquisition. Survey full-scan MS spectra (*m*/*z* 300–1650) were analyzed in the Orbitrap detector with resolution of 35,000 at *m*/*z* 400. The five most intense peptide ions with charge states ≥2 were sequentially isolated to a target value for MS1 of 3 × 10^6^ and fragmented by HCD with a normalized collision energy setting of 25%. The maximum allowed ion accumulation times were 20 ms for full scans and 50 ms for MS/MS, and the target value for MS/MS was set to 1 × 10^6^. The dynamic exclusion time was set to 20 s, and the standard mass spectrometric conditions for all experiments were as follows: spray voltage of 2.4 kV, no sheath, and auxiliary gas flow.

### Data analysis

Acquired RAW data were analyzed by the integrated MaxQuant software v.1.5.2.8, which performed peak list generation and protein identification using the Andromeda search engine [14]. The Uniprot HUMAN_histones 1502 databases was used for peptide identification. Enzyme specificity was set to Arg-C. The estimated false discovery rate of all peptide identifications was set at a maximum of 1%. The mass tolerance was set to 6 ppm for precursor and fragment ions. No missed cleavages were allowed, and the minimum peptide length was set to six amino acids. Variable modifications included lysine D_3_-acetylation (+45.0294 Da); lysine monomethylation (+59.0454, corresponding to the sum of D_3_-acetylation (+45.0294) and monomethylation (+14.016 Da)); dimethylation (+28.031 Da); trimethylation (+42.046 Da); and lysine acetylation (+42.010 Da). To reduce the search time and the rate of false positives, which increase with increasing the number of variable modifications included in the database search [15], the raw data were analyzed through multiple parallel MaxQuant jobs [16], setting different combinations of variable modifications: (1) D_3_-acetylation, lysine monomethylation with D_3_-acetylation, dimethylation, and lysine acetylation, (2) D_3_-acetylation, lysine monomethylation with D_3_-acetylation, dimethylation, and trimethylation, and (3) D_3_-acetylation, lysine monomethylation with D_3_-acetylation, trimethylation, and lysine acetylation. Peptides with Andromeda scores less than 60 and localization probability scores less than 0.75 were removed. Identifications and retention times were used to guide the manual quantification of each modified peptide using QualBrowser version 2.0.7 (ThermoFisher Scientific). Site assignment was evaluated using QualBrowser and MaxQuant Viewer 1.3.0.5. Extracted ion chromatograms (XIC) were constructed for each doubly charged precursor based on its *m/z* value, using a mass tolerance of 10 ppm and a mass precision up to four decimals. For each histone-modified peptide, the percent relative abundance (%RA) was estimated by dividing the area under the curve (AUC) of each modified peptide for the sum of the areas corresponding to all the observed forms of that peptide [17]. For SILAC experiments, Arg10 was selected as heavy label (multiplicity = 2) in MaxQuant. The heavy form of each modified peptide was quantified from its XIC and the % RA calculated. L/H ratios of relative abundances were used to compare samples. To better visualize differences among samples, the ratio of one sample relative to the standard was divided by the average ratios across the samples, obtaining “normalized” ratios, which were visualized and clustered using Perseus [18], with correlation distance and complete linkage as parameters. Differences between luminal A-like and triple negative samples were assessed by *t* test analysis using GraphPad Prism. For statistical analysis, the ratio for peptides quantitated reliably in the spike-in standard but not in the light channel (encircled grey in Fig. 4a) was considered as 0, while those peptides that could not be quantitated in the heavy channel (grey in Fig. 4a), giving an infinite L/H ratio, were not included in the analysis.

## Results

### Evaluation of PAT-H-MS compatibility with histological staining

**Fig. 1:**
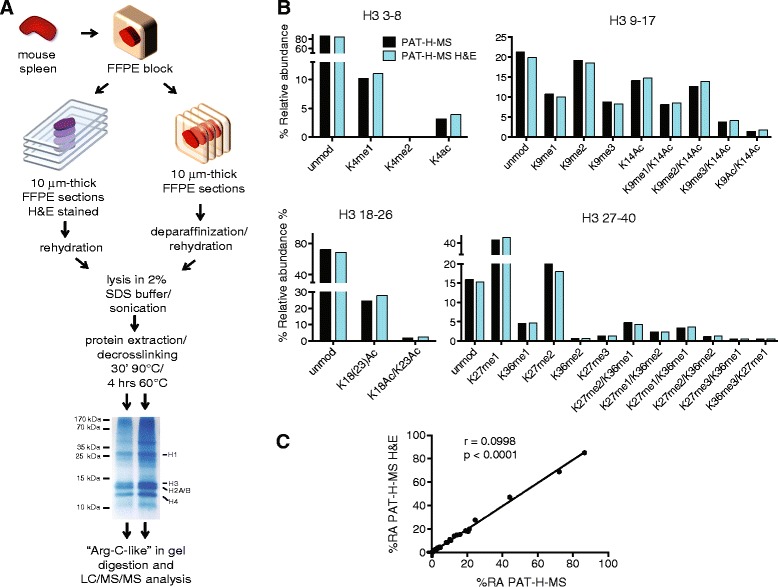
Quantitation of hPTMs by PAT-H-MS in H&E-stained samples. **a** Schematic representation of the procedures used to isolate histones from FFPE mouse spleen using PAT-H-MS on H&E-stained sections compared with the classical approach. **b** Percent relative abundances (%RA) profiles for H3 peptides obtained using the two preparation methods. **c** hPTM %RA correlation for the values shown in **b**. Pearson correlation coefficients (*r*) and *p* values are shown

Tissue sections are usually required to be stained with hematoxylin, alone or coupled with eosin, prior to laser microdissection and other techniques to reduce sample heterogeneity, to better visualize the areas of interest. Therefore, we tested whether the staining procedure can interfere with the PAT-H-MS protocol. To this aim, we performed parallel histone isolations and hPTM analysis from FFPE sections, either stained with hematoxylin and eosin (H&E) with routine staining procedures or left unstained as a control, using mouse spleen as a source of tissue (Fig. 1a). We processed the same starting amount of material-four 10-μm-thick sections-and obtained approximately 80 μg of histone octamer from the classical PAT-H-MS protocol and 40 μg from the H&E-stained sections. Although the yield is lower starting from stained sections, the SDS-PAGE run of the two samples shows very similar protein patterns and purity (Fig. 1a, bottom), and the amount obtained from H&E-stained sections is anyhow largely sufficient for MS-based hPTM analysis, which usually requires 4 μg of histones per run. Importantly, the % relative abundance (%RA) profile for the 25 differentially modified histone H3 peptides that can be analyzed by PAT-H-MS is basically identical using the two extraction methods (Fig. 1b, c), suggesting that histological staining does not affect hPTM detection and profiling.

### PAT-H-MS coupled with manual tissue macrodissection

**Fig. 2:**
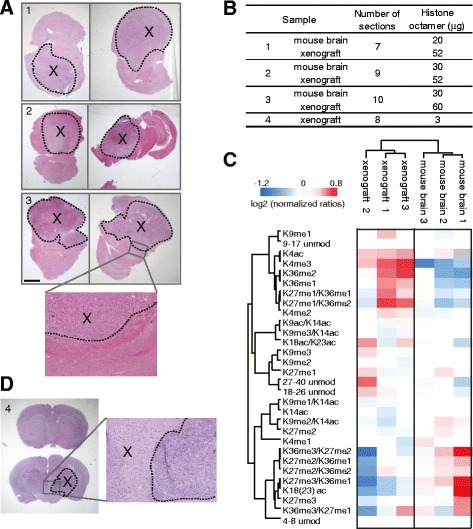
PAT-H-MS coupled with manual macrodissection. **a** H&E staining of mouse brains bearing glioblastoma xenografts (*x*). Scale bar = 2 mm. **b** Number of sections and histone octamer yields for the samples shown in **a** (samples 1–3) and **d** (sample 4). **c** Heatmap display and nonsupervised clustering of the log_2_ of ratios for the indicated hPTMs for macrodissected mouse brains and xenografts from samples 1–3. L/H relative abundances ratios, obtained with the super-SILAC strategy (light channel: breast cancer biopsy, heavy channel: spike-in super-SILAC standard) normalized over the average value across the samples are shown (**d**) H&E staining of a mouse brain bearing a glioblastoma xenografts (*x*) of reduced size

If the area of interest of a tissue section is well defined and sufficiently extended, it can be manually dissected, using a scalpel or razor blade, directly off the tissue sections. Such macrodissection is a relatively quick and inexpensive procedure that can dramatically increase the homogeneity of the tissue. We applied this approach in combination with PAT-H-MS to analyze orthotopic glioblastoma patient-derived xenografts. Because the whole mouse brain, which contains both the normal mouse tissue and the human tumor tissue, was fixed and embedded in paraffin, analyzing the tumor hPTM patterns without any enrichment would not be feasible, thus making this an ideal case to test our method. Since glioblastoma is a very aggressive cancer, the tumor often represents a significant portion of the sample (Fig. 2a), which can be easily identified after H&E staining and manually macrodissected. We analyzed three large xenografts originating from three glioblastoma patients (Fig. 2a), obtaining octamer yields ranging between 50 and 60 μg from seven to ten 10-μm-thick sections (Fig. 2b). As a control, we also macrodissected and included in the analysis the mouse normal brain tissue corresponding to each of the xenografts (Fig. 2a, b). To quantitate more accurately the various histone marks, and particularly the low abundance ones, we used the histone-focused super-SILAC approach that we have recently described, mixing the histone extracted from the FFPE sections with a mix that is used as an internal standard [8, 12]. This strategy originated the SILAC “normalized” ratios (see “Experimental procedures”) displayed in Fig. 2c, which show in human tumor samples a pattern that is clearly different from that found in normal mouse brain, as expected (Fig. 2c, Additional file 1: Dataset S1).

We also sought to apply this strategy to a tumor of reduced size (Fig. 2d). According with the lower amount of starting material, the yield of histone obtained was also lower compared with the other samples (approximately 3 μg (Fig. 2b)). Nevertheless, we were able to quantify all the peptides usually analyzed by PAT-H-MS by using 2 μg of histones from this preparation for digestion and LC-MS/MS analysis (Additional file 1: Dataset S1). This amount of starting material, which roughly corresponds to an area of 8 mm^2^ using ten 10-μm-thick sections, is approaching the lower limit of detection for this approach. The number of sections should be adjusted based on the size of the area of interests, for instance 20 sections should be used for an area that is half the size of the one used here. However, for small tumors or other small features of interest the major limit is represented by the technical capability to manually dissect small areas. Indeed, manual macrodissection is usually adequate only when the area to be dissected is at least a few millimeters in diameter.

### Feasibility of coupling PAT-H-MS with laser microdissection

An alternative to manual macrodissection that provides improved precision is LMD, which allows producing homogeneous tissue cell subpopulations under direct microscopic visualization. LMD has been used in conjunction with many different downstream applications, including global proteomic analyses [19, 20] and the genomic counterpart of our approach, pathology tissue-chromatin immunoprecipitation (PAT-ChIP [21, 22]).

**Fig. 3:**
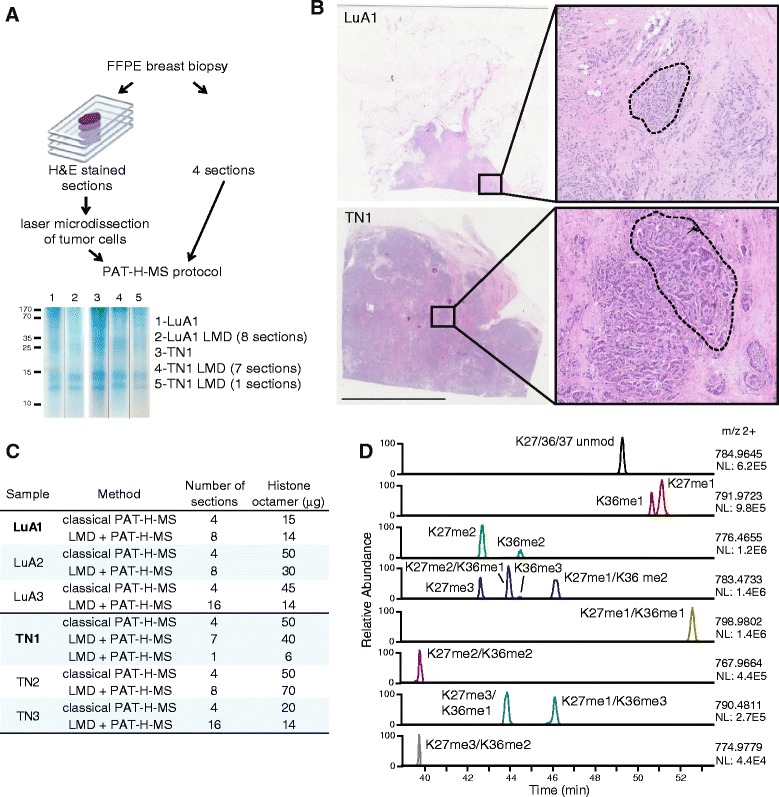
PAT-H-MS coupled with laser microdissection. **a** Schematic representation of the procedures used to isolate histones from FFPE mouse spleen using the classical PAT-H-MS approach (*right*) or LMD-PAT-H-MS (*left*). The appearance of extracted histones using the two procedures is shown for two representative breast cancer samples (LuA1 and TN1). **b** H&E staining for LuA1 and TN1 sections. The *encircled* areas show the microdissected tumor cells. Scale bar = 10 mm. **c** Number of sections and histone octamer yields for six breast cancer luminal A-like (LuA) or triple negative (TN) samples (including the samples LuA1 and TN1 (bold) show in **a** and **b**). **d** Elution profiles of H3-modified (27–40) forms for sample TN1, which was processed using LMD-PAT-H-MS from one section of starting material. Extracted ion chromatograms (XICs) for 14 differentially modified forms are displayed

We recently employed the PAT-H-MS approach to profile hPTM patterns in breast cancer patient samples, revealing significant changes in histone H3 methylation marks between the luminal A-like and triple negative subtypes [8]. In particular, we observed higher levels of K27me3-containing peptides in luminal A-like samples, as already reported in IHC studies [23, 24], and higher levels of K9me3-containing peptides in triple negative samples. For this study, we employed samples having a tumor cellular content of at least 50%, to ensure that a good portion of the sample being analyzed was indeed tumoral. Ideally, though, by selecting only the tumor cells, the detection of more, and more marked, differences is expected. In order to evaluate the feasibility of coupling LMD with our PAT-H-MS approach, we processed three samples belonging to the luminal A-like and triple negative subtypes using our classical approach (the sample had tumor cell content of at least 50%, excluding the adipocytes) or PAT-H-MS coupled with LMD (Fig. 3a-c; Additional file 2: Figure S1). As starting amount, we used four 10-μm-thick sections for the classical approach, while we doubled the number of sections for the LMD samples, to compensate for the reduction of material due to the selection of the tumor cells. When the amount of tumor cells in the slide was particularly low, such as in the case of the LuA3 and TN3 samples, we further increased the number of slides (Fig. 3c). Figure 3a, b shows the Coomassie-stained SDS-PAGE and H&E-stained sections for two representative samples. For all the sample processes, we obtained histones in the amount and purity sufficient for the subsequent LC-MS/MS analysis. Histone yields ranged between 14 and 70 μg (15–50 μg for the classical protocol) and varied mostly based on the amount of tissue present in the sections (Fig. 3b, Additional file 2: Figure S1). To test the lower limit of our approach, for sample TN1 we performed LMD-PAT-H-MS using either one or seven sections. Remarkably, although the amount of histones obtained from one section was lower, as expected, we were able to analyze the whole set of modifications quantifiable by PAT-H-MS (Additional file 3: Dataset S2). As an example of the quality of the results that we obtained from this sample, Fig. 3d shows the extracted ion chromatograms used for peptide quantitation for the 14 differentially modified forms of peptide 27–40, which represents the most complex and challenging H3-modified peptide to profile. While the intensity of the peaks is relatively low, they are very sharp and even low abundance and isobaric peptides can be clearly distinguished and quantitated. The amount of material that was microdissected from one section of sample TN1 corresponds to approximately 450,000 cells, which is the minimum amount of cells that we suggest to use for this type of experiment.

### PAT-H-MS coupled with LMD reveals histone marks distinguishing luminal A-like and triple negative breast cancer patient samples

**Fig. 4:**
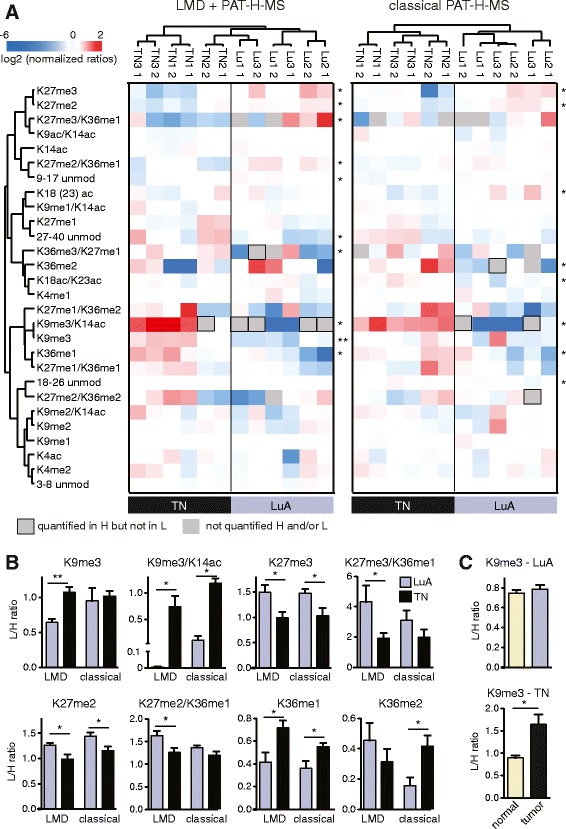
Analysis of luminal A-like and triple negative breast cancer samples by LMD-PAT-H-MS. **a** Heatmap display and nonsupervised clustering of the log_2_ of ratios obtained for the indicated hPTMs for microdissected luminal A-like and triple negative breast cancer samples, using LMD-PAT-H-MS (*left*) or the classical PAT-H-MS approach (*right*). L/H relative abundances ratios obtained with the super-SILAC strategy (light channel: breast cancer biopsy, heavy channel: spike-in super-SILAC standard) normalized over the average value across the samples are shown. Modified peptides significantly different in the two subtypes are indicated by *asterisks*. The *grey* color indicates peptides that were not quantified in the heavy channel or in both heavy and light channels, for which a L/H ratio could not be calculated. *Encircled grey squares* indicate peptides that were not quantified only in the light channel (L/H ratio = 0). **b** Ratios obtained for the indicated peptides in the luminal A-like or triple negative breast cancer samples shown in **a. c** Ratios obtained for the K9me3 peptide in frozen luminal A-like or triple negative breast cancer samples compared with their corresponding normal breast tissue. Five samples were analyzed in the *top panel* and three in the *bottom* one. Samples in **b** and **c** were compared by *t* test. *Error bars* represent standard error from three to six patient samples. *p < 0.05, **p < 0.01

**Fig. 5:**
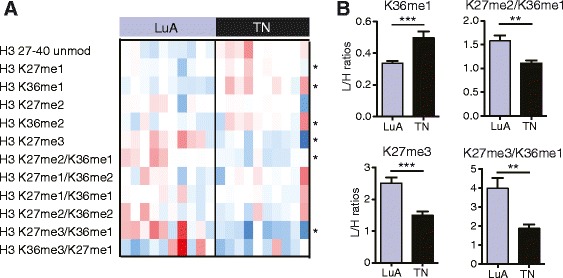
Validation of the K36me1 increase in triple negative breast cancer samples by PAT-H-MS. **a** Heatmap display of the log_2_ of ratios obtained for differentially modified forms of the H3 27–40 peptides in 10 luminal A-like and 10 triple negative breast cancer samples, using the classical PAT-H-MS approach. L/H relative abundances ratios obtained with the super-SILAC strategy (light channel: breast cancer biopsy, heavy channel: spike-in super-SILAC standard) normalized over the average value across the samples are shown. Modified peptides significantly different in the two subtypes are indicated by *asterisks*. **b** Ratios obtained for the indicated peptides in the luminal A-like or triple negative breast cancer samples shown in A. Samples were compared by *t* test. *Error bars* represent standard error from ten patient samples. **p < 0.01, ***p < 0.001

Once demonstrated the technical feasibility of performing PAT-H-MS on LMD samples, we compared the hPTM patterns of the small set of luminal A-like and triple negative samples that we have analyzed, using both the LMD-coupled and classical approaches (Fig. 4). Non-supervised clustering separates luminal A-like and triple negative samples; although, using LMD, TN2 seems to be different from the other two triple negative samples and more similar to luminal A-like samples (Fig. 4a). Some technical variability, demonstrated by the incorrect clustering of some of the technical replicates for the luminal A-like samples, could be observed, but was mostly at the level of low abundance modifications (e.g. K9me3/K14ac) and involved mild differences, that do not impinge on the separation of the two subtypes. Confirming our previous findings [8], we identified several modified peptides that are significantly different in the two groups, including the K27me3- and K9me3-containing peptides. Remarkably, while in the LMD samples we detected significant differences in K9me3, K9me3/K14ac, K27me3, and K27me3/K36me1 peptides, in the samples processed with the classical protocol the difference for the K9me3 and K27me3/K36me1 peptides was not significant (Fig. 4a, b). This is very likely due to the “dilution” effect due to the presence of normal cells within the specimen and is in accordance with the observation that K9me3 levels are higher in tumor cells compared with the surrounding normal cells in triple negative samples, but not in luminal A-like samples (Fig. 4c). A decrease of the K27me2/K36me1 peptide, which we had already observed [8], could also be detected in triple negative samples only with the LMD approach. Interestingly, while the peptide carrying K36me1 in combination with K27me2 decreases, likely due to the contribution of the K27me2 mark, which is also reduced, the K36me1 peptide increases in triple negative samples and could represent an additional biomarker distinguishing luminal A-like and triple negative samples. This novel finding was validated on a larger cohort of 10 luminal A-like and 10 triple negative patients, where the peptide H3 27–40 was profiled by using classical PAT-H-MS approach (Additional file 3: Dataset S2). While corroborating previously found differences, this larger screening also confirmed the increase of the K36me1 mark in triple negative samples (Fig. 5a, b). Few novel significant changes were found in samples processed through the classical approach, but not through LMD-PAT-H-MS, such as an increase in the K36me2 mark. These could reflect changes happening in the normal/non tumoral cells surrounding the tumor, which were excluded by the analysis with LMD, or be randomly detected in this small set of samples due to the heterogeneity of the sections taken as whole. Nevertheless, taken together, these results confirm our hypothesis that by selecting only the tumor cells by LMD, we can detect more marked changes among samples, even in a small dataset such as the one analyzed here and possibly avoid identifying changes due to other tissue components.

## Discussion

In this study, we show the feasibility of applying techniques to reduce sample heterogeneity, including laser microdissection, to the MS-based analysis of hPTM achieved through the PAT-H-MS protocol that we have recently developed [8]. This approach may be useful to separate macroscopic parts of tissues, as exemplified by the isolation of xenografts from mouse brain tissue, or to more finely select specific cell populations, such as tumor cells in a tumor specimen. We compared the classical PAT-H-MS approach with the LDM-coupled strategy, and we showed that while both methods allow distinguishing differences in a small panel of breast tumor samples belonging to different subtypes, LMD-PAT-H-MS allows detecting more marked changes, which results in more significant differences.

However, one issue related to microdissected tissue to consider is the amount of starting material. Whereas for the classical PAT-H-MS approach we found that four 10-μm-thick sections are sufficient for all the >100 samples that we processed so far, laser microdissected samples may require more starting sections, depending on the size of the area of interest to be dissected. For sections containing a large portion of cancerous tissue, such as TN1, one section, corresponding to approximately 450,000 cells, is enough, but when dissecting smaller areas the number of sections should be increased and may become the liming factor of the approach.

Another aspect to consider is the sample size and the labor associated with LMD. Histones can be obtained from FFPE sections using the classical PAT-H-MS approach in approximately 6 h, without the need for any specialized equipment. In the case of the LMD protocol, the dissection time, which is approximately 2 h per section containing 450,000 tumor cells, should be added to the PAT-H-MS processing time. In addition, an LMD instrument and trained personnel are needed.

Therefore, when deciding which approach to use, the benefits and costs of applying such procedures to the analysis of hPTMs should be weighted, taking into account the number of samples, the starting amount available, and the availability of LMD instrumentation. We believe that LDM-PAT-H-MS should represent the approach of choice when dealing with a small sample size for which enough starting material is available, to maximize the differences and the specificity of the results. On the contrary, when processing a large number of samples, the classical PAT-H-MS may be preferred, provided that the samples are chosen to keep tissue heterogeneity to a minimum. For instance, tumor cellularity within a tumor specimen should be 50% or higher. However, in some cases, for instance, when a specific cell subpopulation or a specific morphological feature is needed, LDM-PAT-H-MS is the only alternative available.

Importantly, the analysis of a small cohort of breast cancer confirmed global higher levels of K9me3, and lower levels of K27me3, in triple negative breast cancer samples compared with luminal A-like samples, corroborating our previous results [8] and suggesting possible novel epigenetic biomarkers that may help distinguishing triple negative samples from other breast cancer subtypes. Among these, we have validated on a larger cohort of patients the increase of K36me1, which was not previously reported. Further investigation on this difference, as well as those involving the K27me3 and K9me3 marks, will uncover possible novel epigenetic mechanisms underlying breast cancer.

## Conclusions

Taken together, the results presented in this study show the feasibility of applying techniques to reduce sample heterogeneity, including laser microdissection, to the PAT-H-MS protocol, providing new tools in clinical epigenetics and opening new avenues for the comprehensive analysis of hPTMs in selected cell populations.

## Additional files

Additional file 1: Dataset S1. Dataset containing the measured AUCs for the glioblastoma xenografts and mouse brain samples shown in Fig. 2. (XLSX 33 kb)

Additional file 2: Figure S1. H&E staining of representative sections for the six breast cancer samples analyzed by LMD-PAT-H-MS and classical PAT-H-MS. Scale bar = 10 mm. (PDF 1881 kb)

Additional file 3: Dataset S2. Dataset containing the measured AUCs and L/H ratios for the breast cancer samples shown in Figs. 3, 4, and 5 and Additional file 2: Figure S1. (XLSX 105 kb)

## Abbreviations

FFPE: Formalin-fixed paraffin embedded
H&E: Hematoxylin and eosin
H: Heavy-labeled
hPTMs: Histone post-translational modifications
L: Light-labeled
LMD: Laser microdissection
MS/MS: Tandem mass spectrometry
PAT-H-MS: Pathology tissue analysis of histones by mass spectrometry
RA: Relative abundance
SDS-PAGE: Sodium dodecyl sulphate-polyacrylamide gel electrophoresis
SILAC: stable isotope labeling by amino acid in cell culture
XIC: Extracted ion chromatograms
