# Supplementary figures and images for: PAT-H-MS coupled with laser microdissection to study histone post-translational modifications in selected cell populations from pathology samples
Source: Clin Epigenetics. 2017 Jul 11;9:69. doi: 10.1186/s13148-017-0369-8 (PMC5504751; doi:10.1186/s13148-017-0369-8)

LuA1

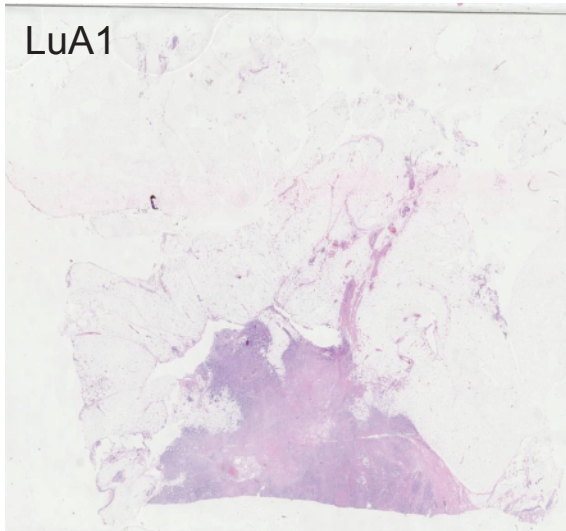

TN1

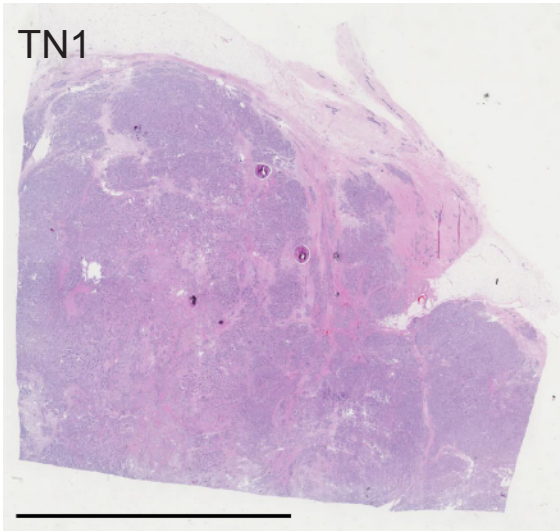

LuA2

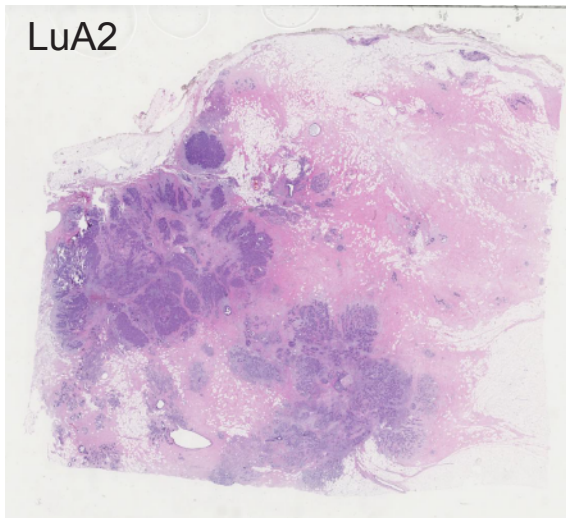

TN2

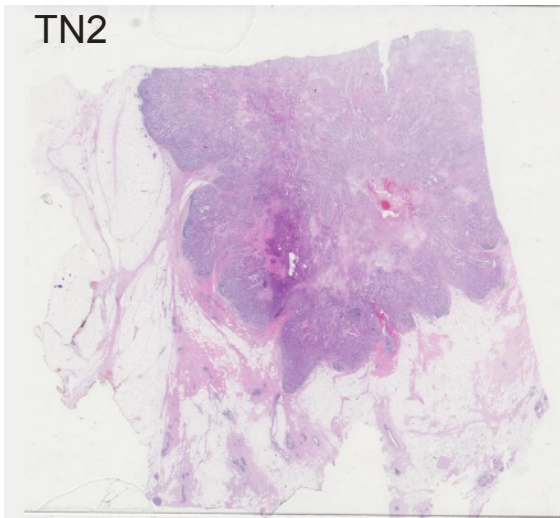

LuA3

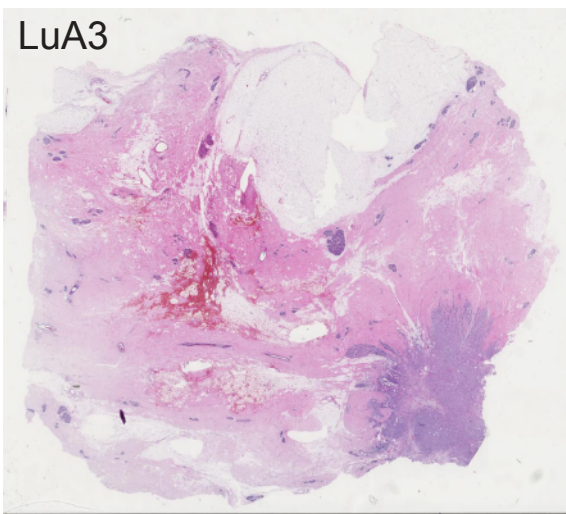

TN3

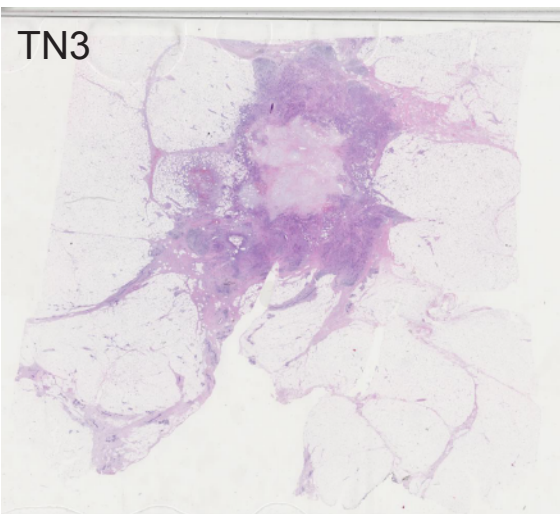

Supplement: Supplementary file 2 — H&E staining of representative sections for the six breast cancer samples analyzed by LMD-PAT-H-MS and classical PAT-H-MS. Scale bar = 10 mm. (PDF 1881 kb) [file 13148_2017_369_MOESM2_ESM.pdf]
